# Injections in hand surgery

**DOI:** 10.1186/1753-6561-9-S3-A2

**Published:** 2015-05-19

**Authors:** Andrew Chin

**Affiliations:** 1 Department of Hand Surgery, Singapore General Hospital, 169608, Singapore

## Introduction

Injection is an essential procedure in medicine, let alone in hand surgery. It entails the insertion of a sharp pointed hollow needle into the body for 2 reasons. Firstly, to retrieve tissue samples from the body and secondly, for the delivery of substances into the body. Injection is thus meant for either diagnostic, therapeutic or both purposes.

Injection provides the hand surgeon with an added option in his/her armamentarium in the management of patient’s condition. It is thus important for the surgeon to be well versed in this procedure that has been proven to be beneficial. A thorough knowledge of the human anatomy and techniques of injection are the important prerequisites in order to achieve success in the use of this tool.

Although it is a minimally invasive procedure, it is not totally safe or innocuous. It has the potential to cause noxious experience for the patient either physically or psychologically. Sometimes the traumatic experience may leave the patient scarred for life and they often develop trypanophobia (fear of injections) and needle phobia subsequently.

Before the advent of proper imaging modalities, the procedure is virtually done blind and hence there is always a risk of potentially damaging structures within the body through trauma during needle insertion. In the current setting where quality imaging with more modalities and options available, injections nowadays can be done under quality imaging guidance thus minimizing or even eliminating the risk of collateral damage within the body.

In addition, injections can now be done to regions of the body that are deeper and less accessible compared to the days when injections were done blind. Appropriate tissues can be obtained, accurate and optimal delivery of therapeutic substances can be achieved leading to better outcomes for the patients.

## Diagnostic purpose

Injections have been used to obtain tissue samples, be it blood, its components and fluid (aspirates), cells (from fine needle) or solid tissue (from needle biopsy). These samples are then sent for further analysis to help the clinician achieve a diagnosis.

Injections can also be used as an adjunct to help diagnosis and subsequent treatment, such as a temporary nerve block to determine if a permanent neurectomy may be effective in treating the cause of pain of a certain region.

## Therapeutic purpose

Delivery of therapeutic substances to the body has been the mainstay for treatment via injection in medicine. From insertion of therapeutic substances through orifices or transdermally as the least invasive modality to open surgery being the most invasive means of delivery of therapeutic substances, delivery of such substances through injection falls in the spectrum between these 2 extremes. There are many ways of delivery of therapeutic substances via injection. Superficially such as into the skin, subcutaneous tissue and muscles or deep such as into body cavities, joints, dural sheath, subarachnoid space, nerve and tendon sheaths, luminal structures such as the blood vessels/hollow organs and solid organs.

Delivery of therapeutic substances via injection usually results in a quicker body response to the substances compared to delivery through the skin and gastro-intestinal tract where absorption through the skin or mucosa usually results in a delay in delivery and response.

Steroid has been a main therapeutic agent that is commonly used in orthopaedics and hand surgery. Together with local anaesthetic agent and given in combination as injectate, it has been used to treat many acute inflammatory conditions of the musculoskeletal system, especially during the early to moderate stages. It is often used also in situations where the patient may not be fit for a more invasive procedure. However, such treatment often provides only temporary relief and may not be curative at all. Fortunately at times, the relief may be long lasting.

## Diagnostic and therapeutic purpose

Aspiration of fluids in the body can be both diagnostic (sample for analysis) and therapeutic (evacuation of fluid as means of decompression).

Nerve blocks can be both diagnostic and therapeutic (pain relief and anaesthesia).

## What’s new

1. Advanced imaging techniques to help surgeon access deep structures accurately within the body to diagnose and to treat.

2. Advancement and discovery of new therapeutic substances such as botulinum toxin, stem cells, plasma rich platelets, growth factors and their derivatives, hyaluronic acid etc. An increase in repertoire of therapeutic agents to help surgeons achieve better patient outcomes.

3. Injection treatment as a viable option over surgery or more invasive modalities for treatment of certain conditions.

